# BISMA - Fast and accurate bisulfite sequencing data analysis of individual clones from unique and repetitive sequences

**DOI:** 10.1186/1471-2105-11-230

**Published:** 2010-05-06

**Authors:** Christian Rohde, Yingying Zhang, Richard Reinhardt, Albert Jeltsch

**Affiliations:** 1 School of Engineering and Science, Jacobs University Bremen, Campus Ring 1, 28725 Bremen, Germany; 2 Max Planck Institute for Molecular Genetics, Ihnestrasse 63-73, D-14195 Berlin-Dahlem, Germany

## Abstract

**Background:**

Bisulfite sequencing is a popular method to analyze DNA methylation patterns at high resolution. A region of interest is targeted by PCR and about 20-50 subcloned DNA molecules are usually analyzed, to determine the methylation status at single CpG sites and molecule resolution.

**Results:**

The BISMA (Bisulfite Sequencing DNA Methylation Analysis) software for analysis of primary bisulfite sequencing data implements sequencing data extraction and enhanced data processing, quality controls, analysis and presentation of the methylation state. It uses an improved strategy for detection of clonal molecules and accurate CpG site detection and it supports for the first time analysis of repetitive sequences.

**Conclusions:**

BISMA works highly automated but still provides the user full control over all steps of the analysis. The BISMA software is freely available as an online tool for academic purposes for the analysis of bisulfite sequencing data from both unique and repetitive sequences http://biochem.jacobs-university.de/BDPC/BISMA/.

## Background

Epigenetic modification of histones and DNA adds heritable information to the genome [1,2]. In mammals, DNA is methylated at the C5 position of cytosine residues mainly in CpG dinucleotides in a tissue specific pattern [3,4]. DNA methylation is an essential process and abnormal methylation is associated with human diseases such as cancer [5,6]. DNA methylation is intensively studied as illustrated by the finding that a PubMed search for 'DNA methylation' retrieved more that 26,000 entries. Bisulfite genomic sequencing is the standard technique for the analysis of DNA methylation at high resolution. In this approach, the genomic DNA is treated with sodium bisulfite, which converts all unmethylated cytosines to uracil, whereas the methylated cytosines remain unconverted. The region of interest is amplified by PCR with primers specific for converted DNA and the PCR product is sequenced [7,8]. Detecting a cytosine in the sequence indicates that the respective position was methylated in the original DNA whereas a thymine indicates that the respective cytosine was unmethylated. When combined with subcloning and sequencing of individual clones, the DNA methylation pattern can be determined at single molecule and nucleotide resolution for continuous tracks of up to 500 base pairs (bps) [9,10].

The analysis of the primary bisulfite sequencing data, which should comprise about 20-50 subcloned DNA molecules for statistical analysis (Additional file 1: Suppl. Text S1), requires the following tasks: 1) the experimental sequences need to be aligned to the *in silico *converted genomic reference. 2) The sequence identity and the conversion rate of each experimental sequence need to be measured, and sequences which do not comply with the quality criteria must be removed. 3) Clonal sequences, which were amplified from the same template molecule in the PCR, need to be detected and removed. 4) The CpG sites need to be identified in the reference sequence and the aligned experimental sequences. 5) The methylation state of the CpG sites in the experimental sequences needs to be determined and the data summarized and presented.

There are different softwares available for the analysis of bisulfite sequencing data, which can be divided into those for analysis of DNA methylation in plant such as Kismeth [11] or mammals such as the BiQ Analyzer [12] and QUMA [13]. While plant methylation analysis conceptually deals with CpG and non-CpG methylation, in mammalian bisulfite sequencing analyses cytosines at non-CpG positions are usually considered an artifact of the method (i.e. incomplete conversion) and used to measure the conversion rate. Here we focus on this approach for bisulfite sequencing data analyses which is assisted by the BiQ Analyzer and QUMA as well. However, both of these have major drawbacks:

• Convenience of use: BiQ Analyzer needs installation and has slow performance.

• Sequence alignment: The BiQ Analyzer sometimes fails to construct the sequence alignment between the experimental and reference sequences without manual user intervention.

• Filtering for clonal sequences: QUMA has not implemented a filtering of clonal sequences. The BiQ Analyzer for certain datasets erroneously suggests removing too many sequences as clonal. In addition, the filtering routine of the BiQ Analyzer software erroneously assumes two molecules as non-clonal, which differ by the existence of an unresolved nucleotide annotation (N-site) only.

• Identification of CpG sites and annotation of methylation states: The BiQ Analyzer often fails detecting CpG sites which are located downstream of a T-stretch. QUMA does not check for the presence of the corresponding CpG guanine in the experimental sequences and annotates methylation states at sites with alignment errors, sequencing errors and mutations, like TA, TT, TN, or CN.

• The genetic diversity among repetitive sequences requires a different strategy for analysis of the bisulfite sequencing data which is not provided by any software so far.

Therefore, we developed a new software called Bisulfite Sequencing DNA Methylation Analysis (BISMA). BISMA provides a highly automated, simple and fast workflow for the analysis of bisulfite sequencing experiments. It can be used for the analysis of subcloned PCR products from unique and repetitive genomic regions. All uploaded data will be automatically processed, filtered for sequence identity, conversion rate and clonal sequences and analyzed. BISMA has implemented an improved strategy for detection of clonal sequences, which preserves identical methylation patterns and ignores N-sites during pattern analysis. Furthermore, BISMA has implemented a new algorithm for detection of CpG sites in the bisulfite sequencing data.

We embedded the BISMA software in the existing DNA methylation data analysis platform BDPC, which allows for further downstream data processing, compilation, web presentation and statistical analysis of results [14] as well as clustering and graphical data presentation [15]. The BISMA software is a freely available online tool for academic purposes http://biochem.jacobs-university.de/BDPC/BISMA/.

## Implementation

### Dataset and software comparison

Biological samples were obtained and treated as described before [16]. Published primary sequencing data of the amplicons 51_new5, 237, 264, 327_III and 335 were used for comparison of the programs [9]. Treatment of cells with 5-azacytidine was performed as described before [9]. Bisulfite treatment, PCR-product purification, subcloning and sequencing was done as described [16].

Briefly, about 200-300 ng genomic DNA were digested with an appropriate restriction enzyme at 37°C overnight to facilitate the subsequent denaturation process, then the DNA was converted with sodium bisulfite in a thermocycler for 15 min at 99°C, 30 min at 50°C, 5 min at 99°C, 1.5 h at 50°C, 5 min at 99°C, 1.5 h at 50°C. The converted DNA was used as a template for PCR. The PCR products were purified by ChargeSwitch PCR Clean-Up Kit (Invitrogen) and subcloned using the StrataClone kit (Stratagene). A high number of clones for each amplicon were sequenced.

The Xist promoter was amplified using the following primers: (FP: GGT AGG GGA ATT AAA AAT GTT TTTT; RP: TAA CCA CTC CTC TTC TAA TCT CTCC) from tailtip DNA of a female mouse. The Alu regions were targeted using the published primer set [17] without the 5'-overhangs (FP: TTT TTA TTA AAA ATA TAA AAA TTA GT; RP: CCA AAC TAA AAT ACA ATAA). The analysis was performed using the matching region of the Alu-Sx subfamily consensus sequence (GeneBank: U14574). We downloaded, installed and used the BiQ Analyzer v2.00 software on a Core Duo L2400 computer with a 1.66 gigahertz processor and 2 gigabyte installed random-access memory on a Microsoft Windows XP professional operation system. We installed JAVA Runtime environment version 6 update 13 (Build 1.6.0_13-b03). We used the remote-ClustalW option such that all multiple sequence alignments were performed on a server at the Max Planck Institute for Informatics. We used 95% and 90% as lower threshold for the conversion rate and the sequence identity in all three programs for comparison.

### BISMA software implementation

BISMA is a PHP coding language based web application which manages the sequencing data information using a MySQL database for temporary storage. Currently, BISMA is running on an openSUSE 10.2 Linux web server. If sequences in ABI file format are uploaded to BISMA the DNA sequence is automatically extracted from the ABI files as they were edited by the user. For this task BISMA uses the Perl module Bio::Trace::ABIF [18]. All pair wise and multiple sequence alignment steps in the BISMA software are performed using the ClustalW software [19,20]. After the alignment, BISMA compares each uploaded sequence with the reference or consensus sequence to calculate the sequence identity, the conversion rate and the occurrence of gaps. Sequences which do not pass the user defined threshold will be excluded from further analysis. BISMA uses the following default quality filtering thresholds for the analysis of unique genomic sequences: sequence identity 90%; conversion rate 95%; gaps 20%. For the analysis of repetitive sequences BISMA uses the following default thresholds: sequence identity 70%; conversion rate: sequences with 3 and more unconverted cytosines in 100 bps are excluded; gaps: 20%.

## Results and Discussion

The BISMA software is designed for upload of primary bisulfite sequencing datasets derived after subcloning of PCR products from unique and repetitive sequences. BISMA has implemented automatically sequence processing, alignment, filtering for sequence quality, filtering of clonal sequences, data analysis and presentation.

### Uploading and sequencing data processing

**Figure 1:**
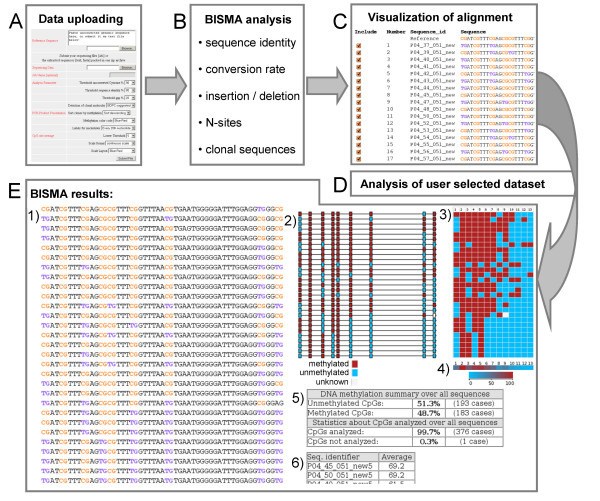
**Workflow of the BISMA software and summary of its result files**. A) Uploading the reference sequence and the bisulfite sequencing data. B) Analysis of the sequencing data by BISMA using the user defined thresholds. Sequences which do not pass the user defined thresholds will be removed. C) Visualization of the alignment of all included sequences. Sequences which pass the filtering for clonal sequences will be pre-selected to be included for later analysis. D) Analysis of the methylation pattern in the user selected dataset. E) All result files can be downloaded in one ZIP file containing: 1) The sequence alignment in which the methylation pattern is highlighted. 2) A graphical representation of the methylation pattern in context of the CpG distribution in the reference sequence. Each DNA sequence is represented by a line and each CpG site by a box. 3) A condensed graphical representation of the methylation pattern. Each row corresponds to one DNA sequence while each column represents a CpG site. 4) A graphical representation of the average methylation at each CpG site. 5) The methylation statistics including the methylation level observed over all sequences and the number of CpG sites that were found to be informative. 6) The methylation levels of the individual sequences.

Sequencing data can either be directly uploaded in ABI file format, as text files containing the extracted sequence or as a single multi-FASTA file (Figure 1A). For the uploading process of separated sequencing files, these files need to be archived using the ZIP standard, which is implemented in current desktop operation systems from Microsoft, Apple and many free Linux distributions. Currently, the total size of all uploaded files is limited to 10 MB, which corresponds to approximately 50 sequences in original ABI format. BISMA automatically prepares two pair wise sequence alignments of the submitted sequence and its reverse complement to the *in silico *converted reference sequence. Based on the quality of the alignments, BISMA determines the correct sequence direction and automatically removes the vector sequence. Pair-wise alignments of the reference sequence with the experimental sequences are used to create an alignment of all sequences. This strategy is significantly faster than a ClustalW multiple sequence alignment and a similar algorithm is implemented in the QUMA software. In principle, such strategy is favorable, since the reference sequence and the experimental sequences of a bisulfite sequencing data set are not of equal weight, but each experimental sequence needs to be compared with the reference sequence.

However, we observed that a ClustalW multiple sequence alignment sometimes will provide a better alignment than the combination of pair-wise alignments. The massive occurrence of thymine after bisulfite conversion and PCR leads to the presence of long poly-T stretches, which often cause PCR and sequencing artifacts in form of thymine insertions or deletions. Under these circumstances the combined pair wise alignments often fail at the last CpG position. Therefore, with default settings BISMA automatically will apply a ClustalW multiple sequence alignment if a CpG site is close to the end of the PCR product and associated with a T-stretch. Beside this, the user can manually choose between pair wise and ClustalW multiple sequence alignment.

### Quality control of aligned sequences

BISMA automatically determines for each sequence the degree of identity to the reference sequence, the conversion rate, the appearance of insertions and deletions and the number of unresolved nucleotides (N-sites) at reference sequence cytosine positions, which are in general an indication of low sequence quality (Figure 1B). These parameters are then used for quality control filtering and sequences which do not pass user defined thresholds will be excluded. Recently, non-CpG methylation has been reported for mammalian cell lines as well which seems to be correlated with the degree of differentiation [21,22]. Although BISMA has a CpG centered approach of data analysis, it can be used for initial visualization of sequences with non-CpG methylation. If this is intended, the threshold of the conversion rate needs to be lowered manually, because otherwise clones with high non-CpG methylation will be excluded because of low conversion. As BISMA does not analyze non-CpG methylation, it might be considered to use the plant methylation analysis softwares Kismeth [11] or CyMATE [23] for this task. However, discrimination of non-CpG methylation from incomplete conversion in mammalian cell types requires careful controls and ideally additional experimental evidence.

### CpG site detection and annotation of the methylation state

**Figure 2:**
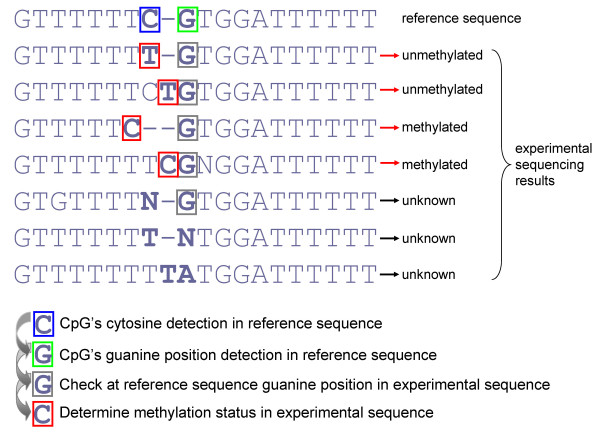
**Improved algorithm for CpG methylation state determination as implemented in the BISMA software**. BISMA detects CpG sites in the reference sequence which is aligned to the experimental sequences. After identification of a guanine at the position aligned to the reference CpG's guanine, the next base in 5' direction is used for determination of the methylation state. Methylation state "unknown" refers to sites lacking clear methylation information due to mutations or sequencing problems.

BISMA uses an improved strategy for detection of CpG site positions in the experimental sequences (Figure 2) when compared to the BiQ Analyzer and QUMA. Briefly, BISMA first checks if a CpG guanine is present in the experimental sequence at the appropriate position and only then determines the methylation state of the CpG site using the sequencing result of the next base in 5' direction.

This strategy allows correct and reliable evaluation of the methylation state and resolves cases of deletions or insertions of thymine by PCR artifacts. As introduced above, such artifacts frequently happen during amplification of T-stretches in the bisulfite converted DNA. BISMA displays the multiple sequence alignment including the annotation of the methylation pattern, unconverted cytosines and possible clonal molecules for visual inspection (Figure 1C).

### Filtering for clonal sequences

**Figure 3:**
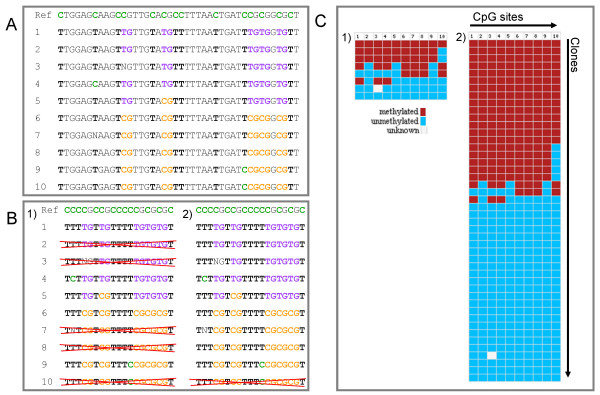
**Improved algorithm for removal of clonal sequences of the BISMA software**. For illustration, a data set obtained in the DNA methylation analysis of in the mouse Xist promoter of a female animal was used, where 50% fully methylated and 50% unmethylated clones are expected. A) Simplified example DNA sequence alignment of bisulfite sequencing data for demonstration of the filtering algorithm. Cytosines in the reference sequence on top of the alignment are indicated in bold green. For the rest of the aligned experimental sequences, methylated CpG sites are highlighted in bold orange, while unmethylated CpG sites are shown in bold purple. Converted cytosines at non-CpG positions are shown in bold black, while conversion artifacts are indicated in bold green. B) Relevant cytosine pattern derived from the multiple sequence alignment includes information about the methylation status of CpG sites and the conversion status of non-CpG site cytosines. 1) Using the strict option BISMA keeps only one sequence out of several with identical patterns. 2) Using the BISMA suggested filtering algorithm only removes clones with identical patterns if these have a conversion artifact at the same position. C) Final methylation pattern obtained after using the 1) strict filtering algorithm or 2) the BISMA suggested filtering algorithm: each square indicates a CpG. Columns represent CpG sites while rows represent single molecules which were subcloned and sequenced. The underlying full DNA sequence alignment of all bisulfite sequencing data is available in Additional file 1: Suppl. Text S9.

One important caveat of bisulfite methylation analysis is the possibility of amplifying single converted DNA molecules ("clonal PCR"), which after subcloning of the PCR product and sequencing of individual clones, could give rise to several identical sequence reads. Therefore, BISMA performs a second filtering to remove such clonal sequences by preparing a pair wise comparison of the cytosine patterns of all sequences (Figure 3A). BISMA ignores N-sites during this procedure, because they indicate bad sequencing data quality and are not suitable to discriminate between cytosine patterns. If two sequences have the same cytosine pattern, these molecules might be either the result of clonal PCR or represent an identical methylation pattern in different template molecules. Removing all sequences with an identical cytosine pattern, therefore, may lead to the erroneous elimination of clones with identical methylation patterns (Figure 3B). This potential mistake is critical in highly methylated or unmethylated PCR products. In contrast, identical patterns of methylation and unconverted cytosines in non-CpG context, which in human DNA usually are considered bisulfite conversion artifacts, always is a clear indication of clonal PCR. Therefore, BISMA removes such sequences with default settings, but it keeps those sequences where the identical cytosine pattern is restricted to CpG sites. However, the user can change the mode of filtering of clonal sequences and manually edit the selection of sequences to be included in the final analysis (Figure 1D). It should be noticed, however, that there is no absolute safe way for filtering of clonal molecules; in critical cases the PCR reaction on the bisulfite converted DNA has to be repeated to resolve this issue experimentally.

### Final output of analyzed data

The BISMA output (Figure 1E) consists of the following elements: 1) an annotated alignment, 2) a graphical display of the methylation pattern in context of the CpG site distribution in the reference sequence, 3) a graphical display of the condensed methylation pattern, 4) a graphical display of the average methylation at each CpG site, 5) statistics about the overall methylation percentage and the percentage of CpG sites analyzed and 6) the overall methylation percentage of each clone. BISMA will automatically sort the sequences in the alignment and all output files and graphical presentations by their methylation level, if selected by the user. BISMA has implemented a threshold for the average methylation report of individual CpG sites, such that only statistics are calculated and reported if at least 5 experimental sequences could be analyzed at the respective site. This avoids over-interpretation and propagation of weak data points. All statistics and the alignment are combined in one HTML file for data storage, presentation and further manual or automatic downstream analysis. For example, the output files of many analyses can be combined and submitted to the BDPC compilation software on the same server.

### Methylation analysis of bisulfite sequencing data from repetitive sequences

The methylation state of repetitive genomic sequences is frequently investigated using bisulfite genomic sequencing as a measurement of global methylation difference or to determine the repeat specific methylation (Additional file 1: Suppl. Text S2). Different approaches have to be used for the investigation of the methylation state of unique and repetitive genomic sequences, because the genomic reference for a repeat sequence is not defined as these elements are present in many copies with similar but not identical sequences. For example, a single primer pair designed to determine the methylation of Alu sequences will amplify PCR-products from about 15,000 template regions in bisulfite converted human DNA [17]. As a consequence, the genomic origin of each amplified molecule is unknown, such that it cannot be directly compared with a defined reference sequence. Therefore, the standard approach for the analysis of unique sequences, where each CpG position is compared against its genomic reference sequence and the methylation state of each CpG site can be determined, is not an option in the analysis of sequencing data from repetitive genomic sequences. Instead, with repeats a consensus sequence has to be used for sequence alignment and analysis. However, this does not allow distinguishing an unmethylated CpG site from one that is mutated to TG. Furthermore, the experimental sequences may contain additional CpG sites which are not present in the consensus.

**Figure 4:**
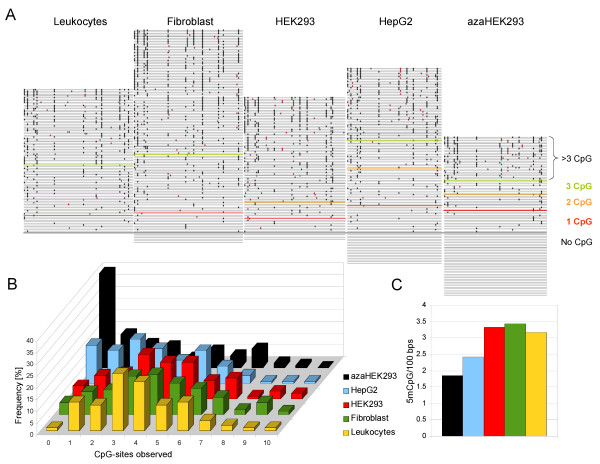
**Analysis of global Alu methylation using the BISMA software for repetitive sequences**. All methylated cytosines in CpG-context are presented and used for calculation. A) Graphical representation of the methylated CpG sites in all sequences of the Alu PCR products at the observed position in the alignment. Each line corresponds to one sequence. The methylated CpGs, which are found at the consensus positions, are represented by black boxes, while those CpGs at other positions are indicated by red boxes. The sequences are sorted according to the total number of methylated CpG. B) Frequency of methylated CpG sites in the sequences from individual clones in different samples. C) Frequency of methylated CpG per 100 bps in all clones in different samples.

To determine the methylation state of repetitive sequences, BISMA has implemented two strategies. The first approach uses the consensus sequence for validation of the amplified sequences and sequence alignment only. The BISMA software for repetitive sequences extracts the position of methylated CpG sites in the aligned experimental sequences and calculates the number of methylated CpG per sequence, which can be located at consensus and non consensus positions. For each sequence, the following information is stored in the output file: the sequence identifier, the number of methylated CpG sites found in the sequence, and the position of each methylated cytosine in the aligned sequence. The methylated cytosines are plotted in an overview graph (Figure 4A). Sequences are sorted according to the number of methylated CpG sites. BISMA visualizes the CpG sites at the consensus sequence positions in black, while those at non consensus positions are plotted in red. BISMA also displays and stores the distribution of methylated CpG sites among sequences (Figure 4B) and the corresponding average occurrence of methylated CpGs in 100 bps (Figure 4C).

To illustrate the functionality of the new BISMA software for analysis of repetitive sequences, we amplified a short region of the Alu repetitive element from bisulfite converted DNA from various samples and used the matching part from the Alu-Sx subfamily consensus sequence [24] for data analysis. As shown in Figure 4, we observed high methylation in the Alu sequences from peripheral blood leukocytes, cultured fibroblast cells, the immortalized cell line HEK293 and the human hepatocellular liver carcinoma cell line HepG2, which is in agreement with literature data on the methylation state of the Alu sequences [17,25,26]. In spite of this, several molecules were found without methylation or low methylation especially in HepG2 and even more pronounced after treatment of HEK293 cells with 5-azacytidine. Such observation is consistent with reported loss of global methylation during cancer progression [6], which for example was documented especially for the Line1 repetitive element in Hep-G2 [27].

As an alternative analysis strategy, we implemented the estimation of the overall methylation as introduced by Yang et al. 2004 [19]. This method focuses on the consensus CpG positions. It estimates the rate of CG to TG mutations in the sequenced strand by determining the number of CG to CA mutations, which corresponds to CG to TG exchanges in the opposite strand. Assuming that the mutation rate is similar in both DNA strands, the number of unmethylated CpG sites can be calculated by correcting the number of TG sites by the fraction of mutated sites. Then, the overall DNA methylation percentage is calculated from the number of unmethylated and methylated CpG positions. Using our test dataset from leukocytes, we obtained an overall methylation of Alu repeats of 93.6%, which is higher than reported previously [17]. Such difference might be in the range of inter-individual variation or result from the absence of the 5'-overhangs in the primers used here.

### Comparison of BISMA with existing bisulfite sequencing analysis programs

We used datasets from published results [9] for the human genes S100B, NCAM2, COL6A2, ZNF295 and H2BFS to compare the performance of the new BISMA software for unique sequences with the BiQ Analyzer and QUMA showing examples of improved CpG site detection and methylation state annotation (Additional file 1: Suppl. Text S3-S5) and filtering for clonal molecules (Additional file 1: Suppl. Text S6-S8).

**Figure 5:**
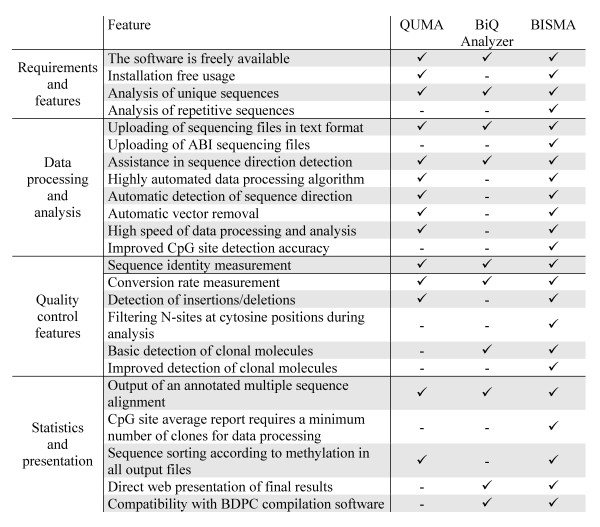
**Comparison of important features of three different programs for analysis of bisulfite sequencing data in a CpG context**.

A summary of the comparison of the BISMA software with the BiQ Analyzer and QUMA software is shown in Figure 5. BISMA is the only software which supports the analysis of bisulfite sequencing data from repetitive sequences. All three programs can be used free of charge for academic purpose. The online tools QUMA and BISMA do not require an installation on the user's computer, while the BiQ Analyzer requires an installation in a JAVA Runtime software environment.

**Figure 6:**
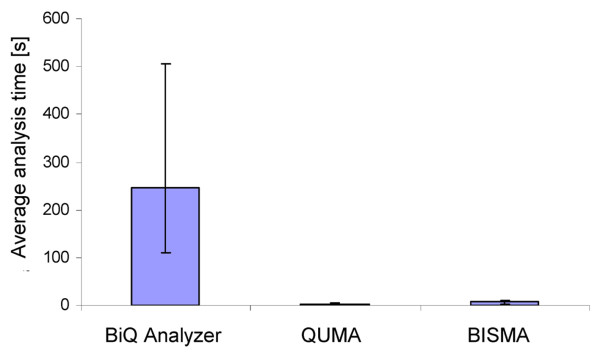
**Average analysis time necessary to process and analyze the example datasets with the BiQ Analyzer, QUMA and BISMA programs**. The bars indicate the lowest and the highest analysis time which was measured.

The result files of the BISMA software for analysis of unique sequences and the BiQ Analyzer are capable for direct web presentation and are compatible with the BDPC compilation software. QUMA and BISMA automatically sort the clones in the multiple sequence alignment, the output files and figures by methylation. For comparison of the analysis time, we measured the time needed for the automated analysis. When comparing the average processing time, QUMA is slightly faster (about 2 fold) than BISMA but the BiQ Analyzer is significantly slower (about 35 fold) (Figure 6). However, QUMA's analysis algorithm has not implemented the filtering of clonal molecules, which might explain the slightly faster performance. The main reason for the gain of speed of the QUMA and BISMA programs when compared with the BiQ Analyzer is the automatic detection of the sequence direction and vector sequence removal, which is implemented in BISMA and QUMA. Another aspect for the increase in speed is the use of pair wise alignments of the reference sequence to the experimental sequences. This strategy is faster than a multiple sequence alignment especially with large number of sequences. A choice between different alignment methods is a unique feature of BISMA.

All three programs support uploading of sequencing files in text format. However, BISMA is the only software which supports direct uploading of ABI sequencing data. All three tools identify and remove improper sequencing results based on sequence identity and conversion rate. In addition, QUMA and BISMA remove sequences with insertions or deletions which do not pass the user defined threshold. Only BISMA directly filters clones for N-sites at cytosine positions as well. All three programs provide an annotated alignment of all sequence of the final dataset.

The accurate determination of the methylation state of each aligned CpG position is one of the most important tasks during analysis of bisulfite sequencing data. The new BISMA software has implemented an improved algorithm for CpG site detection, which detected all positions accurately. The BiQ Analyzer fails if the methylation site under investigation is not directly aligned with the respective cytosine position, which frequently happens in aligned bisulfite sequencing results at T-stretches (Additional file 1: Suppl. Text S3). The QUMA software erroneously reports a methylation result of mutated [TA] and unknown [TN or CN] positions (Additional file 1: Suppl. Text S3-5).

The BiQ Analyzer and BISMA programs offer filtering of clonal molecules, while the QUMA software does not check for clonal sequences and it includes obvious examples of clonal PCR for methylation analysis (Additional file 1: Suppl. Text S6). However, BISMA has implemented an improved algorithm that keeps clones with identical cytosine patterns, which do not have conversion artifacts. More stringent filtering as implemented in the BiQ Analyzer inappropriately removes valid methylation patterns (Suppl. Text S7). BISMA ignores N-sites during filtering of clonal sequences, which is not implemented in the BiQ Analyzer (Additional file 1: Suppl. Text S8). Therefore, the filtering routine of the BiQ Analyzer leads to preferential analysis of sequences with conversion artifacts and bad sequencing data quality. This is critical when analyzing highly methylated or unmethylated regions.

To illustrate the functionality of the improved filtering for clonal sequences, we isolated DNA from mouse tail tip, converted it with sodium bisulfite and amplified a part of the Xist promoter from a female animal (Figure 3C). In females, the Xist promoter is methylated on the active X-chromosome, while unmethylated on the other chromosome which is subject to X-chromosome-inactivation. Therefore, the expected methylation pattern consists of half methylated and half unmethylated clones [28-30]. As shown in Figure 3C, the strict filtering of identical cytosine patterns led to the removal of many clones and artificially created a heterogeneous methylation pattern, which might mislead the interpretation. In contrast, the data analyzed with the modified settings nicely reflect the true methylation pattern.

### BDPC as an integrated platform for bisulfite sequencing DNA methylation analyses

**Figure 7:**
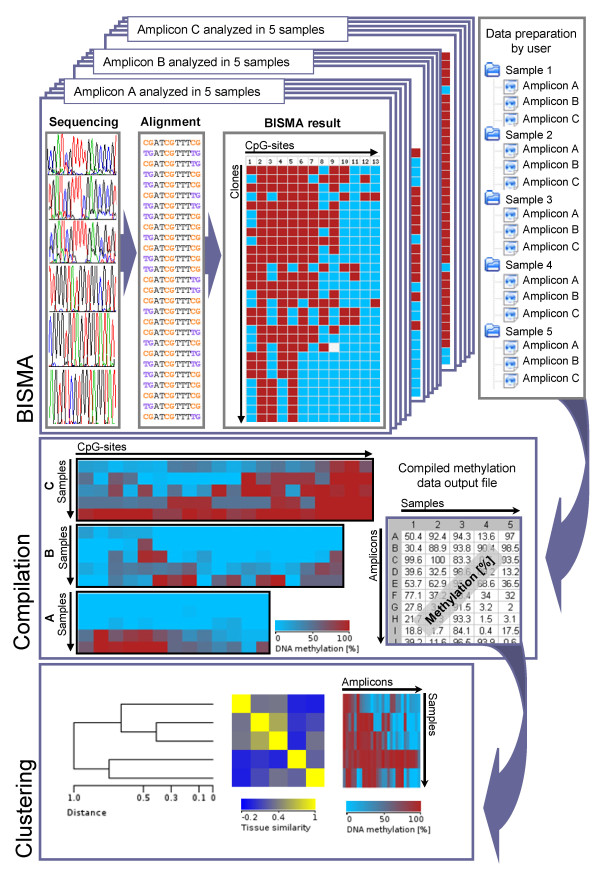
**Integrated bisulfite DNA methylation analysis platform consisting of the BISMA primary sequencing data analysis software and the BDPC compilation and clustering programs**. The result HTML files from BISMA for unique sequences can be further analyzed and displayed with the BDPC compilation software, which will provide an overview table. This table can be used for further result presentations with the BDPC clustering software.

BDPC is a platform, which assists the full DNA methylation analysis workflow (Figure 7). The embedded BISMA software for unique sequences is useful for single PCR product analysis. Its result files can be collected and directly submitted to the BDPC compilation software, which integrates methylation data of CpG sites on the level of amplicons [14]. It displays the whole dataset in form of an HTML presentation, which can be linked to the versatile UCSC genome browser. Finally, large methylation analysis projects often aim to compare the compiled data among the samples analyzed and need condensed pictures for data presentation. These demands are supported by the BDPC clustering software, which prepares a heatmap of all methylation data clustered by the amplicon methylation state and a tissue clustering, which is plotted in a dendrogram [15].

## Conclusions

The BISMA software supports the analysis of subcloned PCR products from bisulfite sequencing experiments. It has implemented an improved strategy for detection of clonal sequences and a new algorithm for detection of CpG sites. BISMA is the first software which supports analysis of bisulfite sequencing from repetitive genomic regions. The highly automated and simple workflow of the BISMA software minimizes the required user interactions to successfully analyze the dataset within a short time. BISMA provides full user control and allows adjusting the thresholds of many parameters and selection of the final dataset on the basis of an annotated sequence alignment. The BISMA software can be freely used as an online tool for academic purposes at http://biochem.jacobs-university.de/BDPC/BISMA/.

## Availability and requirements

**Project name**: BISMA

**Project home page**: http://biochem.jacobs-university.de/BDPC/BISMA/

**Operating system(s)**: Platform independent online software

**License**: Source code available upon request for academic use

**Any restrictions to use by non-academics**: Licence needed

## Abbreviations

BDPC: Bisulfite sequencing Data Presentation and Compilation; BISMA: Bisulfite Sequencing DNA Methylation Analysis software; bps: base pairs; UCSC genome browser: University of California Santa Cruz genome browser; N-sites: unresolved nucleotide sites in sequencing reads

## Authors' contributions

CR and AJ conceived project and wrote the manuscript. CR wrote the BISMA source code. CR, YZ and RR performed the experiments. CR conducted the statistical analyses. All authors read and approved the final manuscript.

## Supplementary Material

Additional file 1 Supplemental Text S1. Correlation between number of analyzed clones and precision of the estimate of biological methylation levels. Supplemental Text S2. Examples in literature for bisulfite sequencing of subcloned PCR-products obtained from repetitive elements. Supplemental Text S3. Improved CpG site detection and improved annotation of the methylation state. Supplemental Text S4. Improved CpG site detection. Supplemental Text S5. Improved annotation of the methylation state. Supplemental Text S6. Automatic detection of clonal molecules. Supplemental Text S7. Improved detection of clonal molecules avoids inappropriate filtering of valid sequences. Supplemental Text S8. Improved detection of clonal molecules ignores N-sites at cytosine positions. Supplemental Figure S9. Complete alignment of all bisulfite sequencing data used to create the results shown in Figure 3C. Click here for file
