# Supplementary material for: BISMA - Fast and accurate bisulfite sequencing data analysis of individual clones from unique and repetitive sequences
Source: BMC Bioinformatics. 2010 May 6;11:230. doi: 10.1186/1471-2105-11-230 (PMC2877691; doi:10.1186/1471-2105-11-230)
Supplement: Additional file 1 — Supplemental Text S1. Correlation between number of analyzed clones and precision of the estimate of biological methylation levels. Supplemental Text S2. Examples in literature for bisulfite sequencing of subcloned PCR-products obtained from repetitive elements. Supplemental Text S3. Improved CpG site detection and improved annotation of the methylation state. Supplemental Text S4. Improved CpG site detection. Supplemental Text S5. Improved annotation of the methylation state. Supplemental Text S6. Automatic detection of clonal molecules. Supplemental Text S7. Improved detection of clonal molecules avoids inappropriate filtering of valid sequences. Supplemental Text S8. Improved detection of clonal molecules ignores N-sites at cytosine positions. Supplemental Figure S9. Complete alignment of all bisulfite sequencing data used to create the results shown in Figure 3C. [file 1471-2105-11-230-S1.PDF]

BISMA - Fast and accurate primary bisulfite sequencing data analysis of individual clones from unique and repetitive sequences

Christian Rohde, Yingying Zhang, Richard Reinhardt, Albert Jeltsch

Additional File 1

- Supplemental Text S1: Correlation between number of analyzed clones and precision of the estimate of biological methylation levels.
- Supplemental Text S2: Examples in literature for bisulfite sequencing of subcloned PCR-products obtained from repetitive elements.
- Supplemental Text S3: Improved CpG site detection and improved annotation of the methylation state
- Supplemental Text S4: Improved CpG site detection.
- Supplemental Text S5: Improved annotation of the methylation state.
- Supplemental Text S6: Automatic detection of clonal molecules.
- Supplemental Text S7: Improved detection of clonal molecules avoids inappropriate filtering of valid sequences.
- Supplemental Text S8: Improved detection of clonal molecules ignores N-sites at cytosine positions.
- Supplemental Figure S9: Complete alignment of all bisulfite sequencing data used to create the results shown in Fig. 3 C.

Supplemental Text S1: Correlation between number of analyzed clones and precision of the estimate of biological methylation levels.

The number of sequences which are used to determine the methylation status of a region and individual CpG sites varies significantly among publications. In the first instance we determined the statistical relevance for a given dataset. When extrapolating the real methylation level of a biological sample from the bisulfite data basic statistics have to be considered. The confidence interval for this estimation is smaller, the more clones were analyzed. This is illustrated below, where for different numbers of analyzed clones, confidence intervals for the real methylation level were calculated assuming an experimental methylation of 50%. Calculations were made using binomial distribution and using a P-value of 0.1. It is not always appreciated, that the statistical error margins are relatively large (experimental uncertainties of course add on top of this). For example, if 6 clones are analyzed and 3 are found methylated, for a P-value of 0.1, the true biological methylation can be estimated to be in between 20-80%. Note, that with smaller P-values, the confidence intervals are getting even larger, in our example it will change to 15-85% for a P-value of 5%. For this reason, one should generally have more than 20 analyzed clones to achieve a reasonable precision for the estimate of the real methylation level.

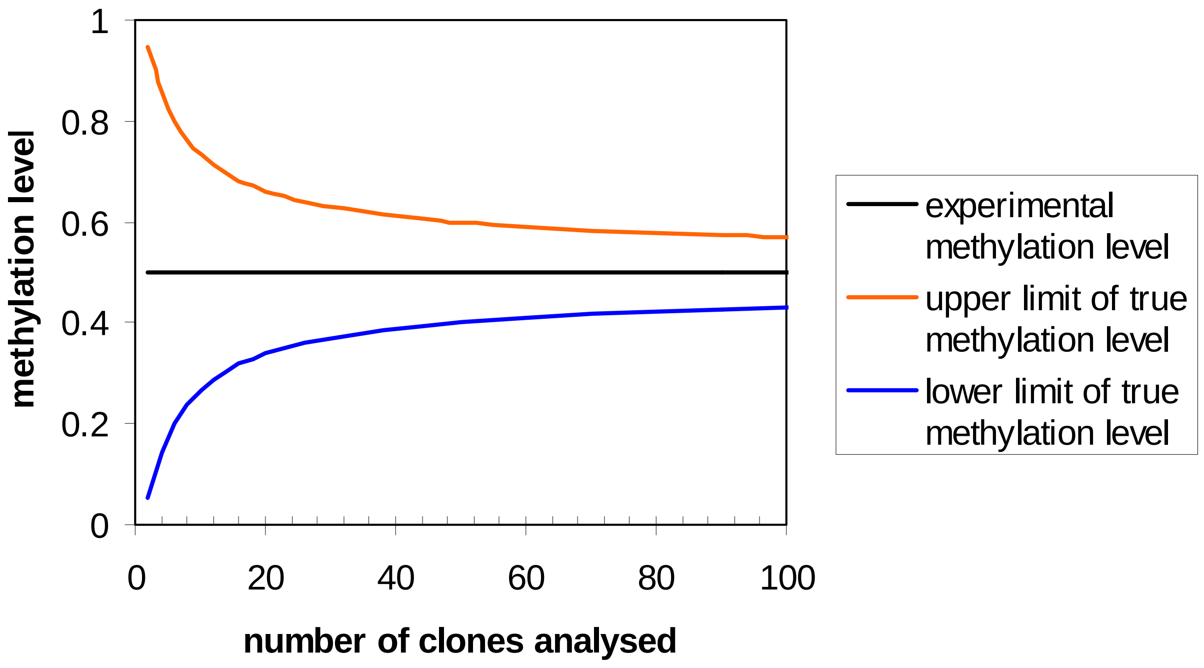

Supplemental Text S2: Examples in literature for bisulfite sequencing of subcloned PCR-products obtained from repetitive elements.

Species	Element	Reference
Mouse	Line-1 (Long Interspersed Nuclear Element-1)	Reuter, 2009; Kuramochi-Miyagawa, 2008; Lane, 2003; Kato, 2007; Hajkova, 2002; Bostick, 2007; Yaman, 2006; Woodcock, 1998; Sharif, 2007; Bian, 2009
	IAP (Intracisternal A Particle)	Kuramochi-Miyagawa, 2008; Lane, 2003; Kim, 2004; Dong, 2008; Kato, 2007; Lucifero, 2007; Kurihara, 2008; Dodge, 2004; Howell, 2001; Hajkova, 2002; Lees-Murdock, 2008; Bostick, 2007; Ramirez, 2006; Yaman, 2006; Sharif, 2007; Yamagata, 2007; Ramirez, 2007; Morita, 2007
	MusD (AL773522)/Etn (Early Transposon)	Kim, 2004; Maksakova, 2009; Dong, 2008;
	MuERV-L (murine endogenous-retrovirus-L)	Ramirez, 2006; Ramirez, 2007;
	MLV (murine leukemia virus)	Dong, 2008
	SineB1	Kato, 2007
	Minor satellite	Kato, 2007; Yamagata, 2007
	Major satellite	Chen, 2003; Kato, 2007; Bian, 2009; Yamagata, 2007; Morita, 2007
	centromeric satellite	Kim, 2004;
Homo sapiens	L1	Yang, 2004; Hansen, 2003; Woodcock, 1997; Perrin, 2007; El-Maarri
	Alu	Cadieux, 2006; Yang, 2004; Perrin, 2007
	Alpha satellite	Gopalakrishnan, 2009
	Satellite 2	Gopalakrishnan, 2009; Cadieux, 2006; Mund, 2005; Hassan, 2001; Kaup, 2006; Perrin, 2007
	Major satellite	Chen, 2004
	3.3-kb repeat	Katargin, 2009
Pig	centromeric satellite (Z75640)	Archer, 2003; Petkov, 2009; Chen 2006; Kang, 2001;
	PRE-1 SINE	Archer, 2003;
Cow	Satellite I	Kang, 2001; Kang, 2002; Wee, 2007; Kang, 2005
	Satellite 2	Kang, 2005
	Alpha satellite	Wee, 2007; Kang, 2005
Rabbit	RsatIIE	Chen, 2004; Chen 2006; Zhang, 2005

1. Archer GS, Dindot S, Friend TH, Walker S, Zaunbrecher G, Lawhorn B, Piedrahita JA: **Hierarchical phenotypic and epigenetic variation in cloned swine**. *Biology of reproduction* 2003, **69**(2):430-436.
2. Bian Y, Alberio R, Allegrucci C, Campbell KH, Johnson AD: **Epigenetic marks in somatic chromatin are remodelled to resemble pluripotent nuclei by amphibian oocyte extracts**. *Epigenetics* 2009, **4**(3):194-202.
3. Bostick M, Kim JK, Esteve PO, Clark A, Pradhan S, Jacobsen SE: **UHRF1 plays a role in maintaining DNA methylation in mammalian cells**. *Science (New York, NY* 2007, **317**(5845):1760-1764.
4. Cadieux B, Ching TT, VandenBerg SR, Costello JF: **Genome-wide hypomethylation in human glioblastomas associated with specific copy number alteration, methylenetetrahydrofolate reductase allele status, and increased proliferation**. *Cancer research* 2006, **66**(17):8469-8476.
5. Chen T, Tsujimoto N, Li E: **The PWWP domain of Dnmt3a and Dnmt3b is required for**

directing DNA methylation to the major satellite repeats at pericentric heterochromatin. *Molecular and cellular biology* 2004, **24**(20):9048-9058.
6. Chen T, Zhang YL, Jiang Y, Liu JH, Schatten H, Chen DY, Sun QY: **Interspecies nuclear transfer reveals that demethylation of specific repetitive sequences is determined by recipient ooplasm but not by donor intrinsic property in cloned embryos**. *Molecular reproduction and development* 2006, **73**(3):313-317.
7. Chen T, Zhang YL, Jiang Y, Liu SZ, Schatten H, Chen DY, Sun QY: **The DNA methylation events in normal and cloned rabbit embryos**. *FEBS letters* 2004, **578**(1-2):69-72.
8. Dodge JE, Kang YK, Beppu H, Lei H, Li E: **Histone H3-K9 methyltransferase ESET is essential for early development**. *Molecular and cellular biology* 2004, **24**(6):2478-2486.
9. Dong KB, Maksakova IA, Mohn F, Leung D, Appanah R, Lee S, Yang HW, Lam LL, Mager DL, Schubeler D *et al*: **DNA methylation in ES cells requires the lysine methyltransferase G9a but not its catalytic activity**. *The EMBO journal* 2008, **27**(20):2691-2701.
10. El-Maarri O, Becker T, Junen J, Manzoor SS, Diaz-Lacava A, Schwaab R, Wienker T, Oldenburg J: **Gender specific differences in levels of DNA methylation at selected loci from human total blood: a tendency toward higher methylation levels in males**. *Human genetics* 2007, **122**(5):505-514.
11. Gopalakrishnan S, Sullivan BA, Trazzi S, Della Valle G, Robertson KD: **DNMT3B interacts with constitutive centromere protein CENP-C to modulate DNA methylation and the histone code at centromeric regions**. *Human molecular genetics* 2009.
12. Hajkova P, Erhardt S, Lane N, Haaf T, El-Maarri O, Reik W, Walter J, Surani MA: **Epigenetic reprogramming in mouse primordial germ cells**. *Mechanisms of development* 2002, **117**(1-2):15-23.
13. Hansen RS: **X inactivation-specific methylation of LINE-1 elements by DNMT3B: implications for the Lyon repeat hypothesis**. *Human molecular genetics* 2003, **12**(19):2559-2567.
14. Hassan KM, Norwood T, Gimelli G, Gartler SM, Hansen RS: **Satellite 2 methylation patterns in normal and ICF syndrome cells and association of hypomethylation with advanced replication**. *Human genetics* 2001, **109**(4):452-462.
15. Howell CY, Bestor TH, Ding F, Latham KE, Mertineit C, Trasler JM, Chaillet JR: **Genomic imprinting disrupted by a maternal effect mutation in the Dnmt1 gene**. *Cell* 2001, **104**(6):829-838.
16. Kang YK, Koo DB, Park JS, Choi YH, Kim HN, Chang WK, Lee KK, Han YM: **Typical demethylation events in cloned pig embryos. Clues on species-specific differences in epigenetic reprogramming of a cloned donor genome**. *The Journal of biological chemistry* 2001, **276**(43):39980-39984.
17. Kang YK, Koo DB, Park JS, Choi YH, Lee KK, Han YM: **Influence of oocyte nuclei on demethylation of donor genome in cloned bovine embryos**. *FEBS letters* 2001, **499**(1-2):55-58.
18. Kang YK, Lee HJ, Shim JJ, Yeo S, Kim SH, Koo DB, Lee KK, Beyhan Z, First NL, Han YM: **Varied patterns of DNA methylation change between different satellite regions in bovine preimplantation development**. *Molecular reproduction and development* 2005, **71**(1):29-35.
19. Kang YK, Park JS, Koo DB, Choi YH, Kim SU, Lee KK, Han YM: **Limited demethylation leaves mosaic-type methylation states in cloned bovine pre-implantation embryos**. *The EMBO journal* 2002, **21**(5):1092-1100.
20. Katargin AN, Pavlova LS, Kisseljov FL, Kisseljova NP: **Hypermethylation of genomic 3.3-kb repeats is frequent event in HPV-positive cervical cancer**. *BMC medical genomics* 2009, **2**(1):30.
21. Kato Y, Kaneda M, Hata K, Kumaki K, Hisano M, Kohara Y, Okano M, Li E, Nozaki M, Sasaki H: **Role of the Dnmt3 family in de novo methylation of imprinted and repetitive sequences during male germ cell development in the mouse**. *Human molecular genetics* 2007,

16(19):2272-2280.
22. Kaup S, Grandjean V, Mukherjee R, Kapoor A, Keyes E, Seymour CB, Mothersill CE, Schofield PN: **Radiation-induced genomic instability is associated with DNA methylation changes in cultured human keratinocytes**. *Mutation research* 2006, **597**(1-2):87-97.
23. Kim SH, Kang YK, Koo DB, Kang MJ, Moon SJ, Lee KK, Han YM: **Differential DNA methylation reprogramming of various repetitive sequences in mouse preimplantation embryos**. *Biochemical and biophysical research communications* 2004, **324**(1):58-63.
24. Kuramochi-Miyagawa S, Watanabe T, Gotoh K, Totoki Y, Toyoda A, Ikawa M, Asada N, Kojima K, Yamaguchi Y, Ijiri TW *et al*: **DNA methylation of retrotransposon genes is regulated by Piwi family members MILI and MIWI2 in murine fetal testes**. *Genes & development* 2008, **22**(7):908-917.
25. Kurihara Y, Kawamura Y, Uchijima Y, Amamo T, Kobayashi H, Asano T, Kurihara H: **Maintenance of genomic methylation patterns during preimplantation development requires the somatic form of DNA methyltransferase 1**. *Developmental biology* 2008, **313**(1):335-346.
26. Lane N, Dean W, Erhardt S, Hajkova P, Surani A, Walter J, Reik W: **Resistance of IAPs to methylation reprogramming may provide a mechanism for epigenetic inheritance in the mouse**. *Genesis* 2003, **35**(2):88-93.
27. Lees-Murdock DJ, Lau HT, Castrillon DH, De Felici M, Walsh CP: **DNA methyltransferase loading, but not de novo methylation, is an oocyte-autonomous process stimulated by SCF signalling**. *Developmental biology* 2008, **321**(1):238-250.
28. Lucifero D, La Salle S, Bourc'his D, Martel J, Bestor TH, Trasler JM: **Coordinate regulation of DNA methyltransferase expression during oogenesis**. *BMC developmental biology* 2007, **7**:36.
29. Maksakova IA, Zhang Y, Mager DL: **Preferential epigenetic suppression of the autonomous MusD over the nonautonomous ETn mouse retrotransposons**. *Molecular and cellular biology* 2009, **29**(9):2456-2468.
30. Morita S, Horii T, Kimura M, Goto Y, Ochiya T, Hatada I: **One Argonaute family member, Eif2c2 (Ago2), is essential for development and appears not to be involved in DNA methylation**. *Genomics* 2007, **89**(6):687-696.
31. Mund C, Hackanson B, Stresemann C, Lubbert M, Lyko F: **Characterization of DNA demethylation effects induced by 5-Aza-2'-deoxycytidine in patients with myelodysplastic syndrome**. *Cancer research* 2005, **65**(16):7086-7090.
32. Perrin D, Ballestar E, Fraga MF, Frappart L, Esteller M, Guerin JF, Dante R: **Specific hypermethylation of LINE-1 elements during abnormal overgrowth and differentiation of human placenta**. *Oncogene* 2007, **26**(17):2518-2524.
33. Petkov SG, Reh WA, Anderson GB: **Methylation changes in porcine primordial germ cells**. *Molecular reproduction and development* 2009, **76**(1):22-30.
34. Ramirez MA, Pericuesta E, Fernandez-Gonzalez R, Moreira P, Pintado B, Gutierrez-Adan A: **Transcriptional and post-transcriptional regulation of retrotransposons IAP and MuERV-L affect pluripotency of mice ES cells**. *Reprod Biol Endocrinol* 2006, **4**:55.
35. Ramirez MA, Pericuesta E, Fernandez-Gonzalez R, Pintado B, Gutierrez-Adan A: **Inadvertent presence of pluripotent cells in monolayers derived from differentiated embryoid bodies**. *The International journal of developmental biology* 2007, **51**(5):397-407.
36. Reuter M, Chuma S, Tanaka T, Franz T, Stark A, Pillai RS: **Loss of the Mili-interacting Tudor domain-containing protein-1 activates transposons and alters the Mili-associated small RNA profile**. *Nature structural & molecular biology* 2009.
37. Sharif J, Muto M, Takebayashi S, Suetake I, Iwamatsu A, Endo TA, Shinga J, Mizutani-Koseki Y, Toyoda T, Okamura K *et al*: **The SRA protein Np95 mediates epigenetic inheritance by recruiting Dnmt1 to methylated DNA**. *Nature* 2007, **450**(7171):908-912.
38. Wee G, Shim JJ, Koo DB, Chae JI, Lee KK, Han YM: **Epigenetic alteration of the donor cells does not recapitulate the reprogramming of DNA methylation in cloned embryos**.

Reproduction (Cambridge, England) 2007, **134**(6):781-787.
39. Woodcock DM, Lawler CB, Linsenmeyer ME, Doherty JP, Warren WD: **Asymmetric methylation in the hypermethylated CpG promoter region of the human L1 retrotransposon**. *The Journal of biological chemistry* 1997, **272**(12):7810-7816.
40. Woodcock DM, Linsenmeyer ME, Warren WD: **DNA methylation in mouse A-repeats in DNA methyltransferase-knockout ES cells and in normal cells determined by bisulfite genomic sequencing**. *Gene* 1998, **206**(1):63-67.
41. Yamagata K, Yamazaki T, Miki H, Ogonuki N, Inoue K, Ogura A, Baba T: **Centromeric DNA hypomethylation as an epigenetic signature discriminates between germ and somatic cell lineages**. *Developmental biology* 2007, **312**(1):419-426.
42. Yaman R, Grandjean V: **Timing of entry of meiosis depends on a mark generated by DNA methyltransferase 3a in testis**. *Molecular reproduction and development* 2006, **73**(3):390-397.
43. Yang AS, Estecio MR, Doshi K, Kondo Y, Tajara EH, Issa JP: **A simple method for estimating global DNA methylation using bisulfite PCR of repetitive DNA elements**. *Nucleic acids research* 2004, **32**(3):e38.

Supplemental Text S3: Improved CpG site detection and improved annotation of the methylation state

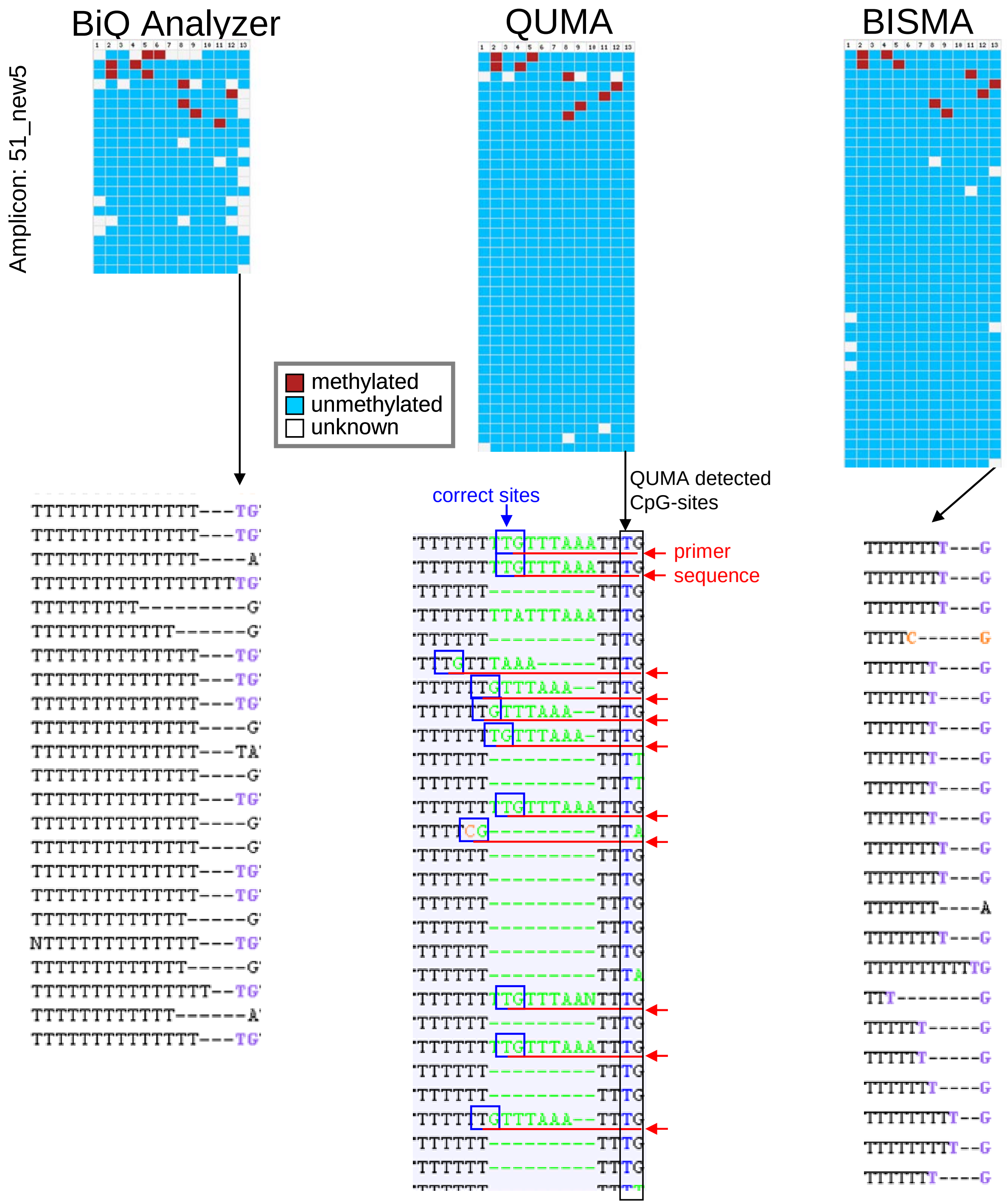

- BISMA correctly processes the data
- QUMA aligns wrong positions and annotates primer TG as unmethylated. QUMA fails detecting several CpG sites
- BiQ Analyzer fails detecting CpG sites next to a T-Stretch

Supplemental Text S4: Improved CpG site detection.

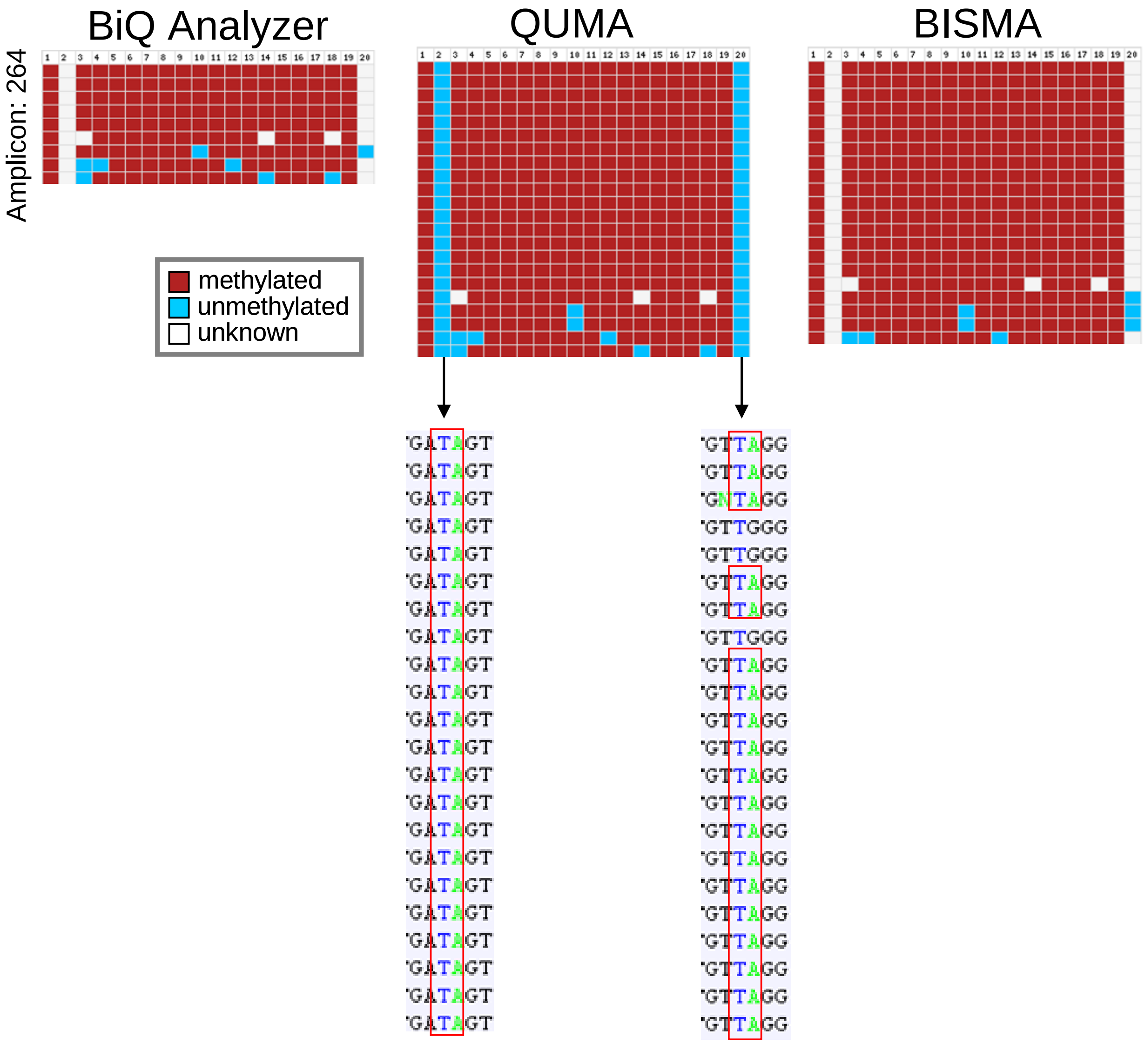

- BISMA correctly processes the data
- QUMA reports mutated CpG positions (TA) as unmethylated
- BiQ Analyzer correctly determines the methylation status, but erroneously removes several clones with the same methylation pattern as clonal

Supplemental Text S5: Improved annotation of the methylation state.

QUMA software multiple sequence alignment:

51_new4 CpG_13 | **335** CpG_8, CpG_10 | **Xist** CpG_3, CpG_7 | **237** CpG_27, CpG_33

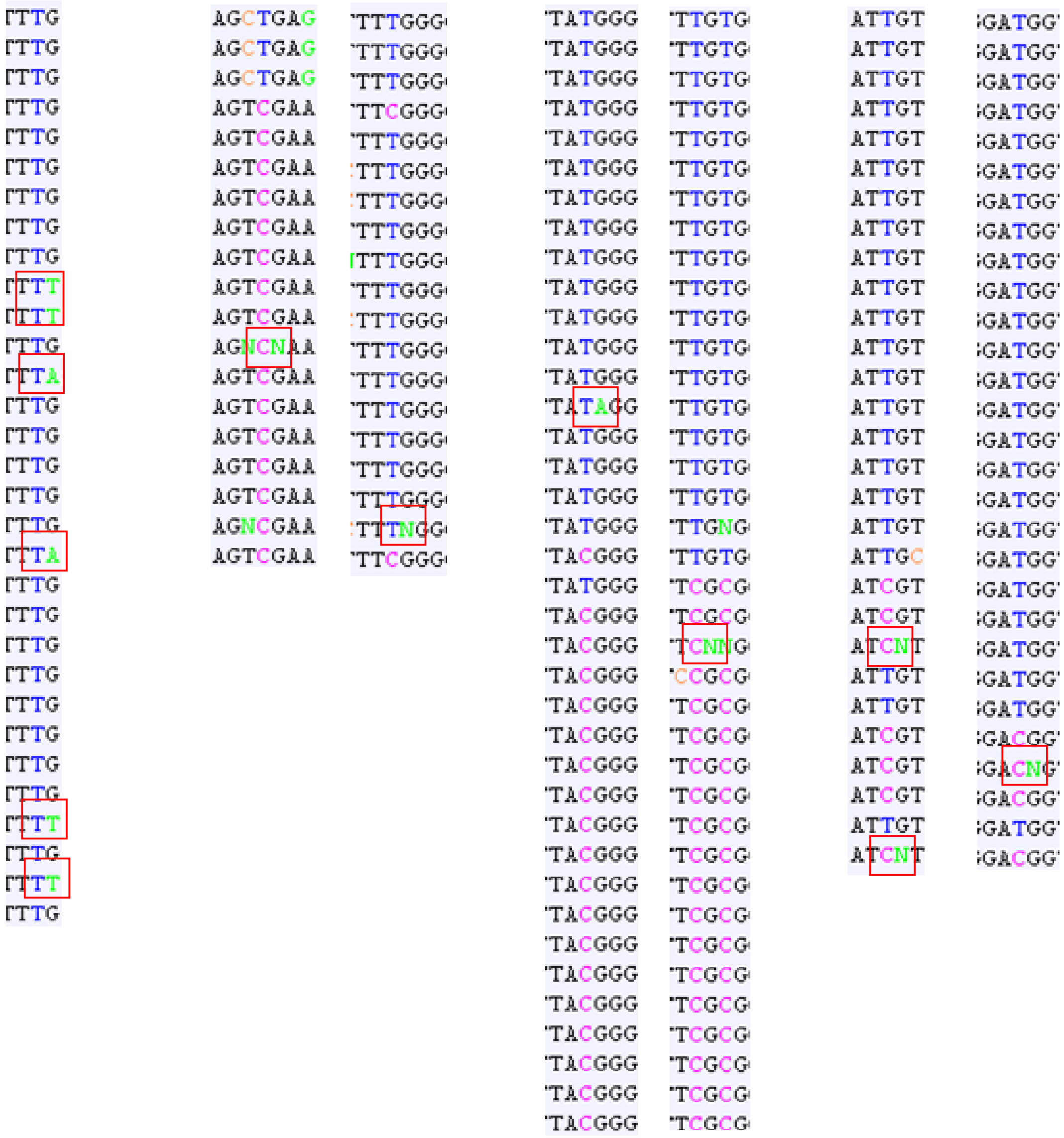

- QUMA reports methylation state of TT and TA sites as unmethylated
- QUMA reports CpG positions with unknown status (TN and CN) as unmethylated or methylated

Supplemental Text S6: Automatic detection of clonal molecules.

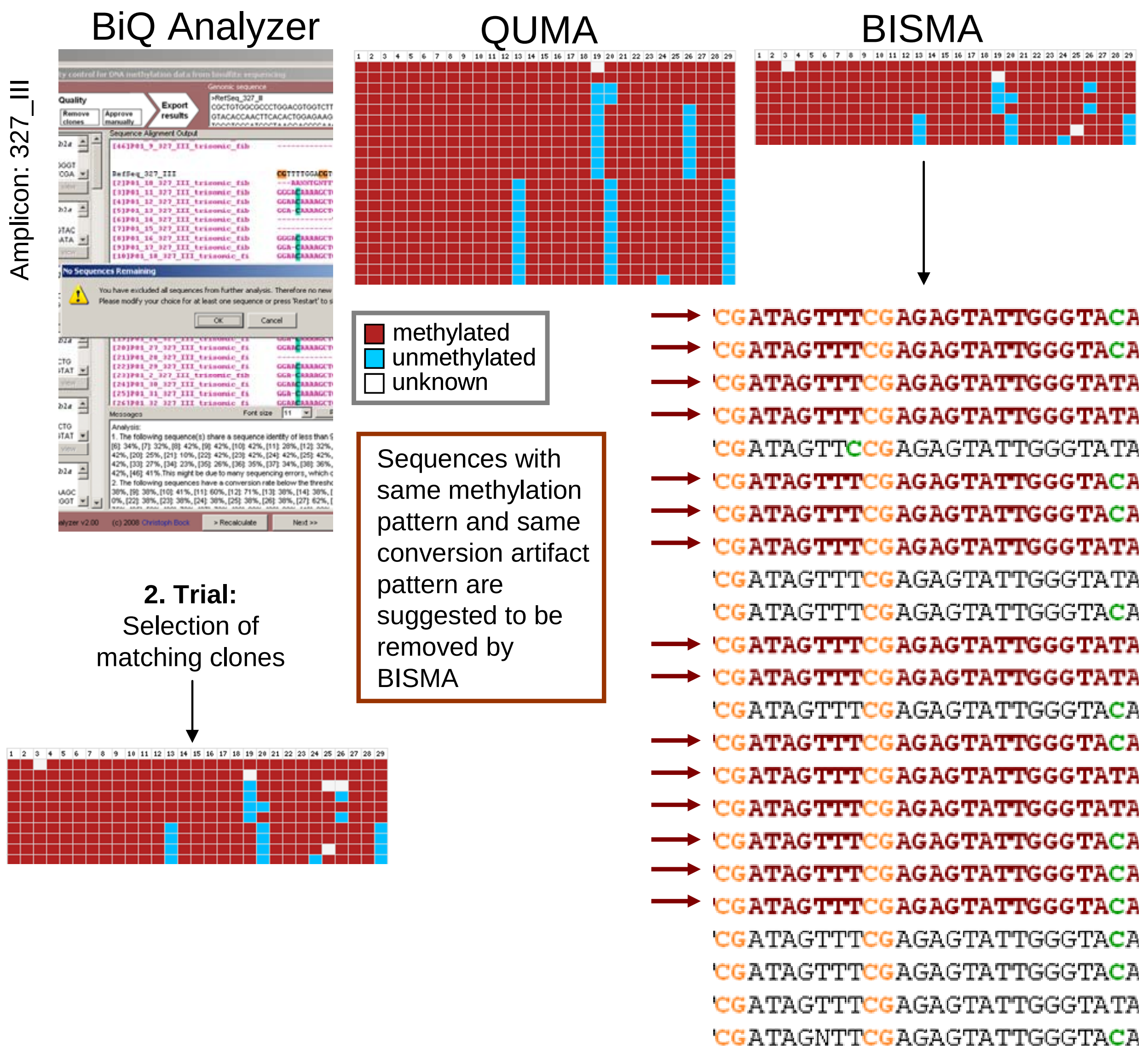

- BISMA correctly processes the data
- QUMA has not implemented a filtering of clonal molecules
- BiQ Analyzer fails to process data automatically. Only if a selection of matching clones is submitted to BiQ Analyzer, it manages the analysis and filters most of the clonal molecules in this dataset. Two clonal sequences are erroneously included, because they contain N-sites at the CpG position (for a more detailed example about this issue cf. S7).

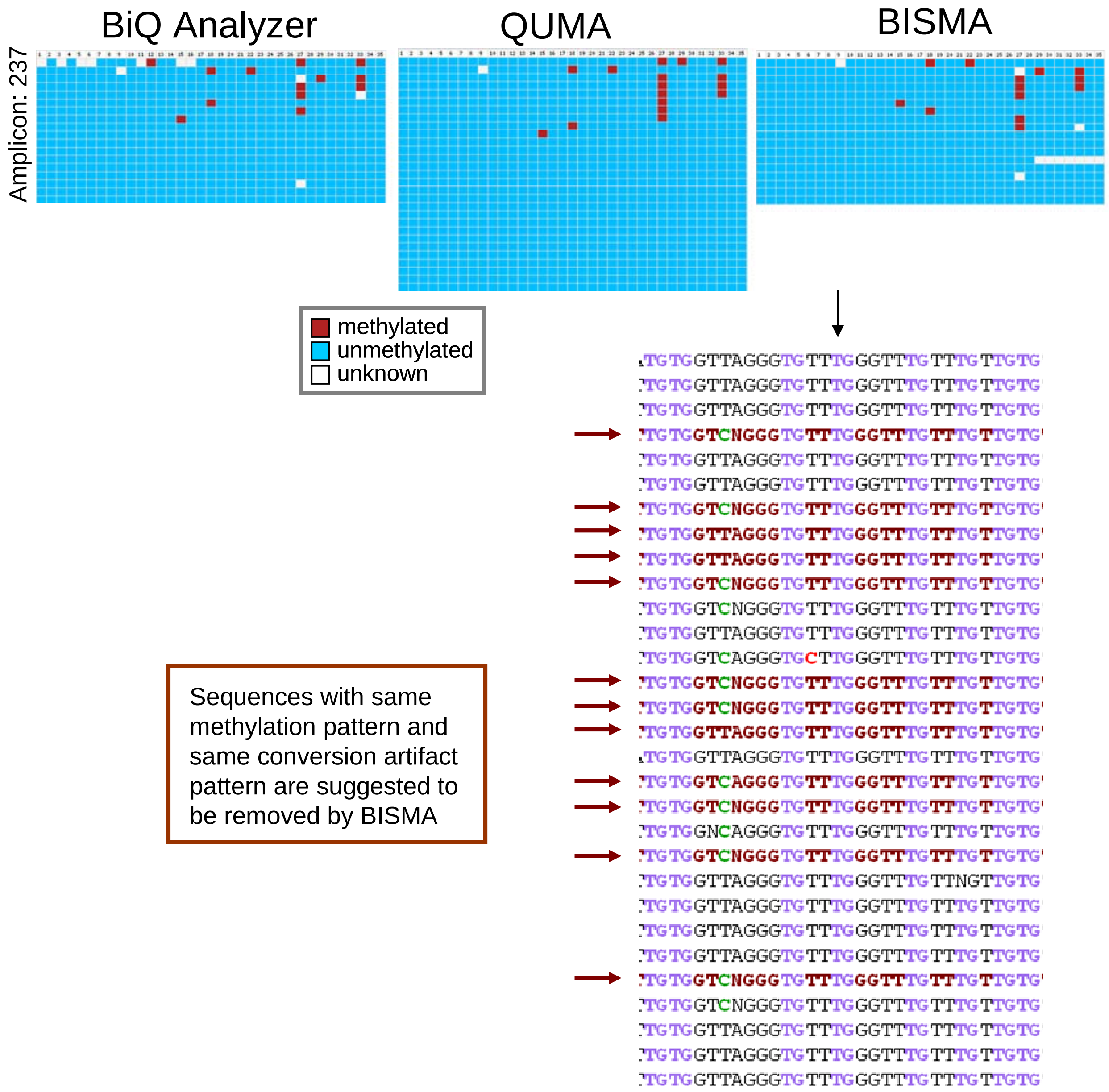

- BISMA correctly processes the data
- QUMA has not implemented a filtering of clonal molecules
- BiQ Analyzer correctly processes the data

Supplemental Text S7: Improved Algorithm for detection of clonal molecules avoids inappropriate filtering of valid sequences.

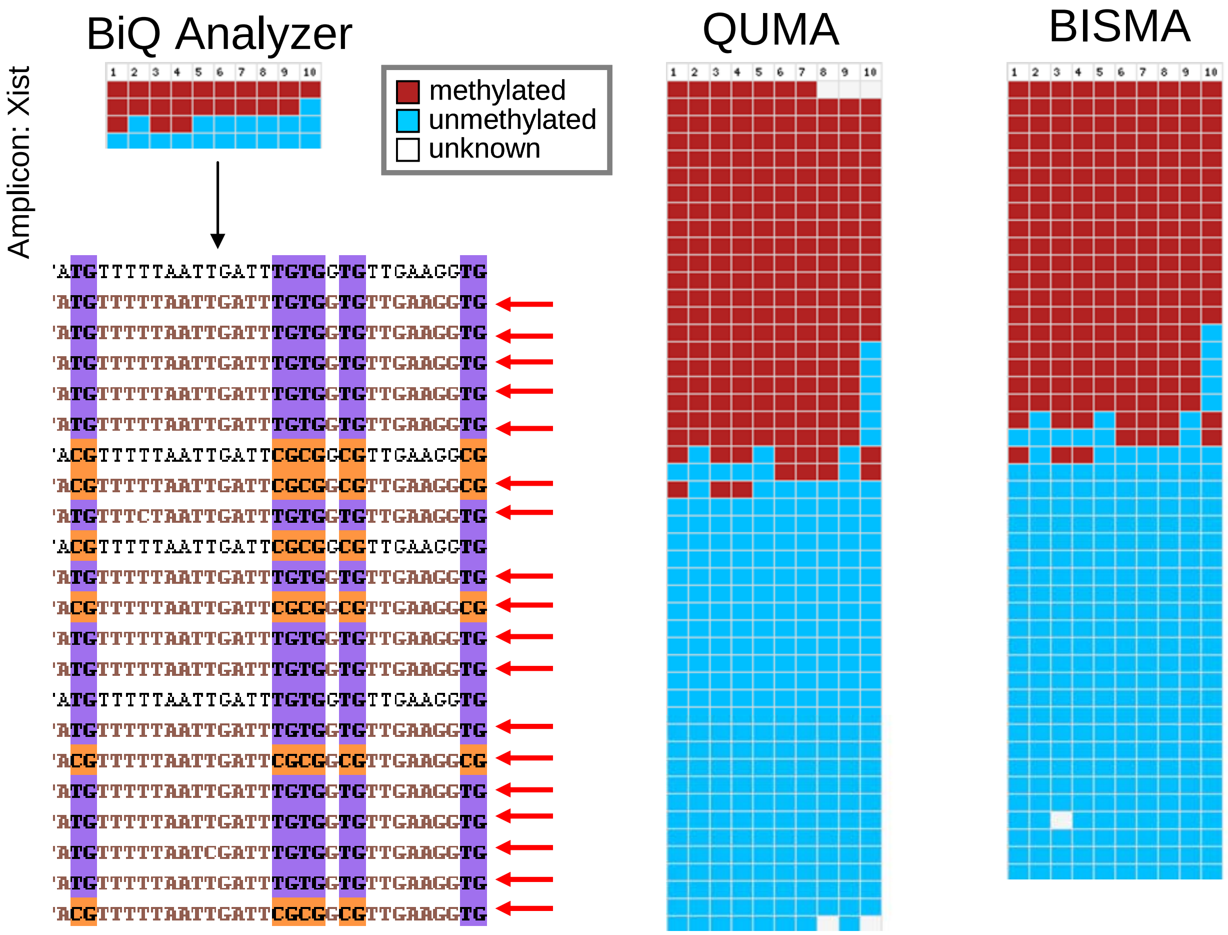

- BISMA correctly processes the data
- QUMA does not filter clonal molecules
- BiQ Analyzer suggests removing all data with the same methylation pattern (indicated with red arrows) in absence of conversion artifacts

Supplemental Text S8: Improved Algorithm for detection of clonal molecules ignores N-sites at cytosine positions.

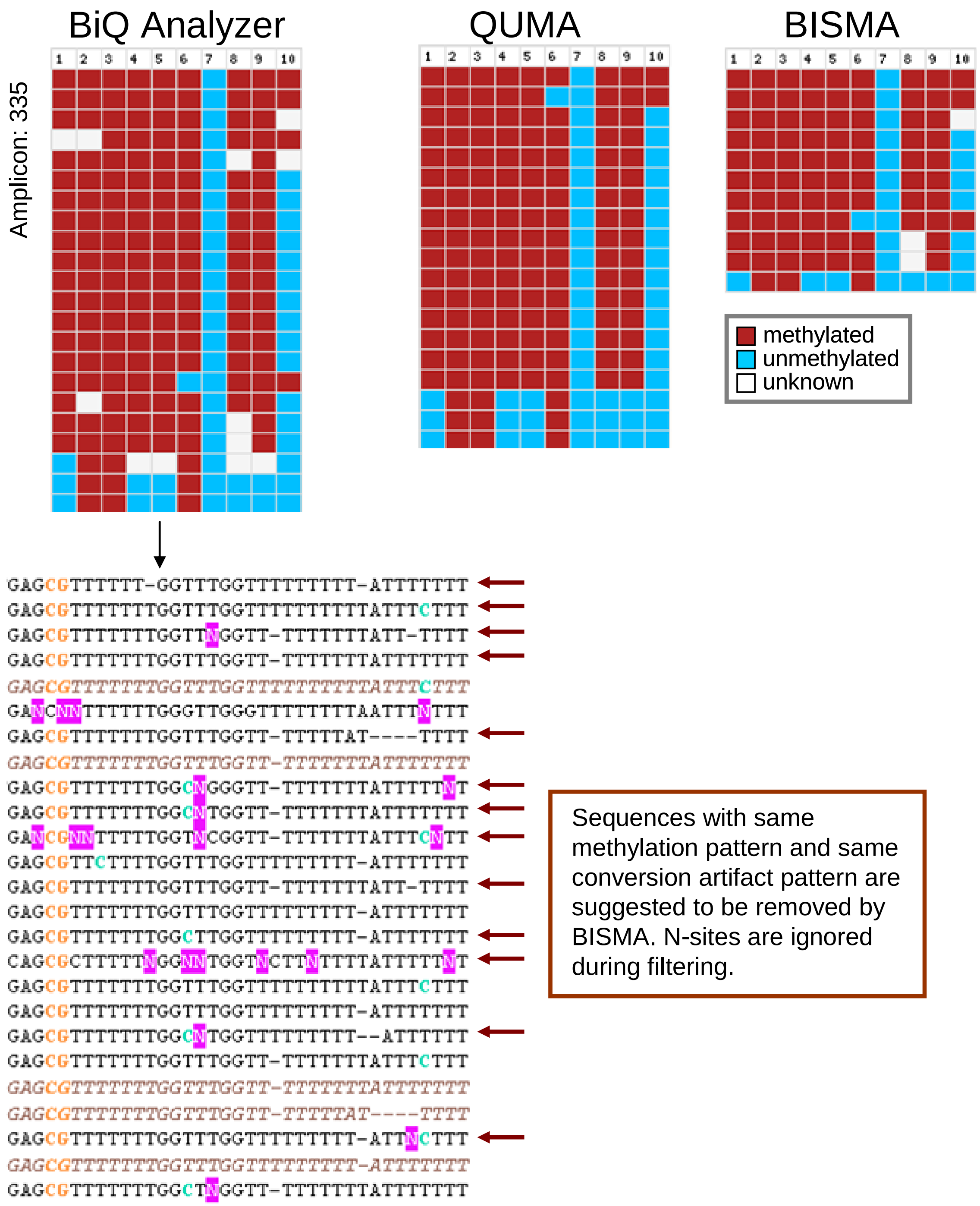

- BISMA correctly processes the data
- N-sites disrupt the filtering of clonal molecules in the BiQ Analyzer
- QUMA has not implemented a filtering of clonal molecules

Supplemental figure S9: Complete alignment of all bisulfite sequencing data used to create the results shown in Fig. 3 C

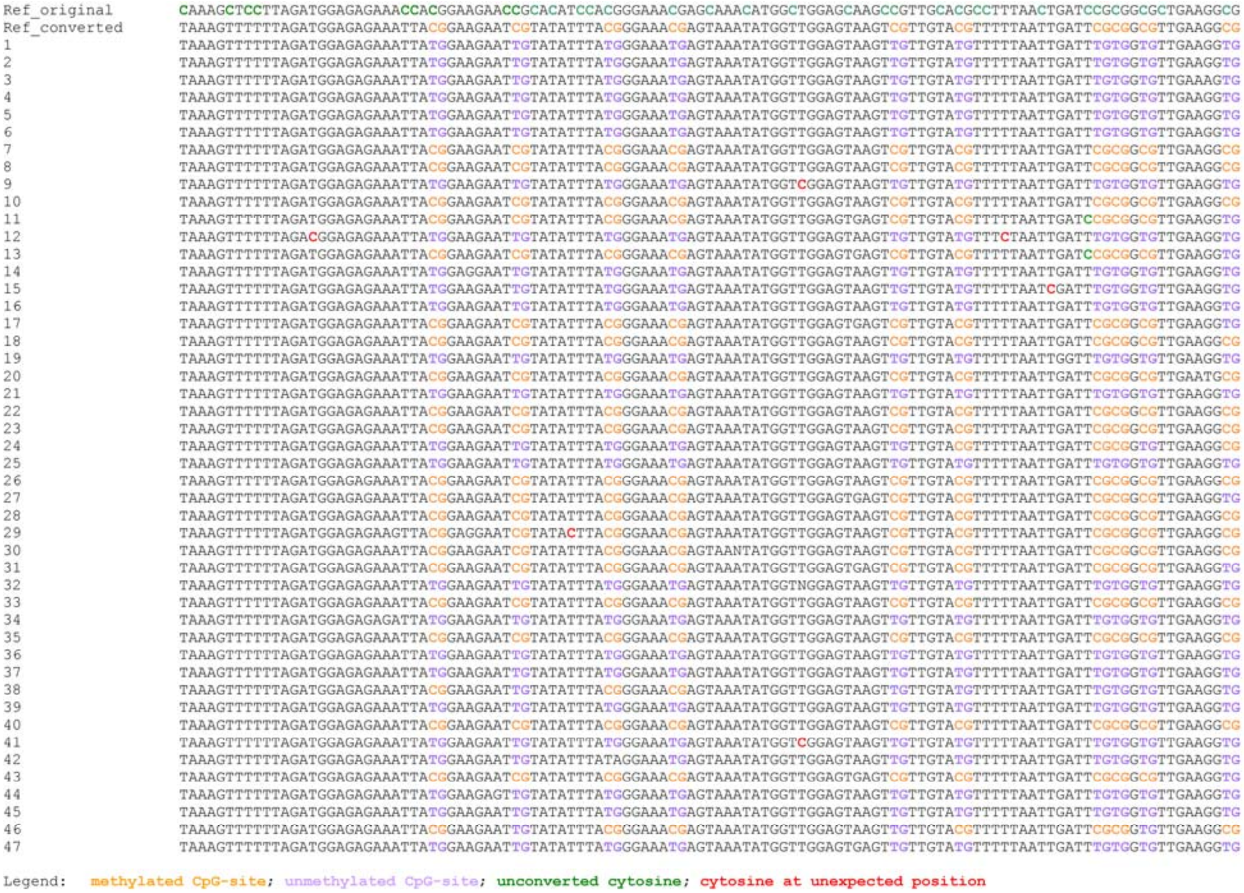

Cytosines in the reference sequence on top of the alignment are indicated in bold green. For the rest of the aligned experimental sequences, methylated CpG sites are highlighted in bold yellow, while unmethylated CpG sites are shown in bold purple. Converted cytosines at non CpG-positions are shown in bold black, while conversion artifacts are indicated in bold green.
